# Giant Hepatic Cysts: Prenatal Findings And Uncommon Postnatal Outcome

**Published:** 2012-04-01

**Authors:** F Sauvat, L Harper, F Cuillier, T Abossolo, J.L Alessandri, J.L Michel

**Affiliations:** Department of Pediatric Surgery, CHR Reunion, Saint Denis, Reunion; 1 Department of Obstetrics and Fetal Medicine, CHR Reunion, Saint Denis, Reunion.; 2 Department of Neonatal Intensive Care, CHR Reunion, Saint Denis, Reunion.

**Keywords:** hepatic cysts, antenatal diagnosis

## Abstract

With modern prenatal imaging, liver cysts are being diagnosed more often. Although large cysts are usually asymptomatic, they may present as an abdominal emergency requiring surgery in the first weeks of life. We report a series of 3 patients with prenatal diagnosis of isolated cystic liver lesions diagnosed at 22, 31 and 33 weeks of gestational age. The hepatic origin of the cysts was confirmed prenatally by a MRI in 2 cases, with visualization of a normal gallbladder. The prenatal course was uneventful. Postnatal ultrasound confirmed the diagnosis of liver cyst, showed normality of the biliary tract and in one case, rupture of the cyst during delivery. Because of an uncommon rapid increase in size, the 3 children underwent surgical excision of the cysts within the first weeks of life. These were non-bile-containing intrahepatic cysts arising from segment IV. Long-term follow up was uneventful.

## INTRODUCTION

Improvements in fetal ultrasound (US) have increased the accuracy of prenatal diagnosis of congenital liver and biliary malformations. There are relatively few reports in the literature about prenatal diagnosis of isolated liver cysts [1-3]. Clinicians dealing with such fetuses are presented with the difficult task of prenatal counseling concerning neonatal management. Usually, if a liver cyst is suspected, it is of paramount importance to differentiate this lesion from other cysts, which require prompt neonatal treatment (such as duodenal duplications or biliary atresia). Liver cysts are considered to be of good prognosis, especially if there is prenatal visualization of a normal gallbladder thus excluding the diagnosis of biliary atresia [4]. We report a series of 3 fetuses with prenatal diagnosis of isolated non-bile-containing intrahepatic cyst (NBIC), which presented unusual postnatal growth requiring surgery within the first weeks of life.

## CASE SERIES

**Patient 1 **(Fig. 1):

A 25-year-old woman, with no past medical history was referred to our tertiary care hospital following diagnosis of an abdominal cystic lesion in her male fetus. Her previous ultrasound (US) performed at 22 weeks gestation was normal. The US realized at 31st week of pregnancy showed an isolated anechoic cyst of 43x21mm, on the inferior part of liver. MRI confirmed the liver cyst. Spontaneous delivery occurred at 37 weeks. Postnatal US and CT (computed tomography) showed ascites due to rupture of the cyst. Spontaneous resolution during the first month of life was good with normal clinical findings, but at the age of six weeks, the baby presented with signs of acute abdomen (pain, ascites) requiring emergency percutaneous aspiration followed by surgery because of cyst recurrence. Laparotomy confirmed the diagnosis of a NBIC in segment IV and marsupialization was performed.

**Figure:**
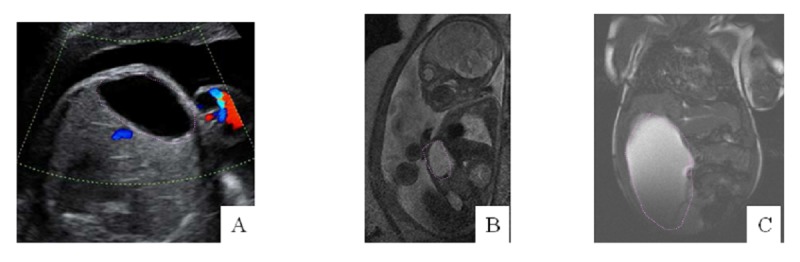
Figure 1: Patient 1 imaging: prenatal US (A) at 31 weeks of gestation with discovery of anechogenic lesion forward the liver, confirmed by MRI (B). Postnatal MRI (C) (at 6 weeks of age) attesting rapid cyst growing.

**Patient 2 **(Fig. 2):

A 34-year-old patient was referred to our institution following prenatal diagnosis at 33 weeks of pregnancy of a liver lesion in her male fetus. Our US confirmed a cystic lesion of 75x65 mm, arising from the right aspect of the liver with no vascularization abnormalities and normal biliary tract. CT-scan performed on the second day of life confirmed the liver cyst and surgery was performed on day 7 because of the size of the lesion. Marsupialization of a NBIC arising from right lobe was performed.

**Figure:**
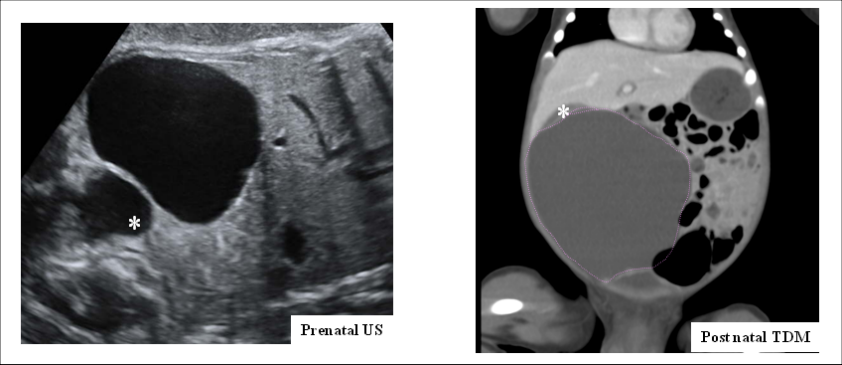
Figure 2: Patient 2 imaging: Prenatal US showing a large intra-hepatic cyst, and a normal gladbladder (*). CT scann at births confirmed a very large hepatic cyst and the normal gladbladder (*).

**Patient 3** (Fig. 3):

A 38- year-old patient was referred to us following prenatal diagnosis at 22 weeks gestation of a cystic lesion developed under and behind the right part of the liver. The lesion seemed to communicate with the gallbladder on ultrasound and initially a diagnosis of choledochal cyst was first envisaged. MRI confirmed the presence of a cystic lesion of right liver lobe as well as the lack of communication between the cyst and the biliary tract. The prenatal diagnosis was then revised to that of a liver cyst. Postnatal US confirmed the diagnosis of liver cyst and showed rapid growth and complete excision of the lesion was hence performed at 6 weeks of life because of digestive symptoms due to compression by the cyst. The NBIC was located in segment IV.

**Figure:**
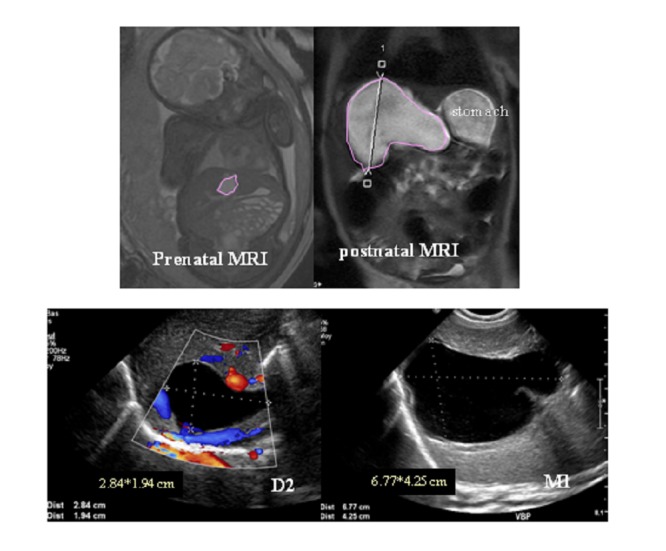
Figure 3: Patient 3 imaging: prenatal MRI confirmed hepatic cyst located in segment IV and postnatal evolution in MRI realized preoperatively at 6 weeks of life. Postnatal US illustrated the rapid growing of hepatic cyst between days 2 of life (D2) and the first months of life (M1).

In these three cases, histology confirmed the diagnosis of NBIC with a unilocular cyst with largely cuboidal epithelial lining and an underlying loose connective tissue. Long-term outcome was uneventful at 2 years follow-up with no recurrences, even in the first two cases that underwent only marsupialization.

## DISCUSSION

Prenatal diagnosis of congenital liver cyst is rare and their incidence is unknown (for example, in a 10-year Australian study, 3 hepatic cysts were identified in 22,000 births) [5]. The prevalence of simple liver cysts in adults is about 3-18% [6]. Their etiology is unknown, and it is believed that they arise from aberrant bile ducts either by an inflammatory or obstructive process or by vascular disruption [7].

As in our experience, NBICs are usually unilocular, and are characterized by cuboidal epithelium with no connection to the normal biliary tree [3]. The diagnosis of NBIC can be envisaged in presence of an anechogenic cyst surrounded by liver parenchyma on prenatal US. Doppler sonography can be of use by showing the relation between the cyst and the portal vessels and hepatic artery. Presence on US of septa within the cyst is usually regarded as a marker for other pathologies such as mesenchymal hamartomas.

The differential diagnosis of these anechoic intrahepatic cysts includes choledochal cysts, biliary atresia (usually small cyst within the hilum, with a very small or absent gallbladder) or duodenal duplication [4]. If there are multiples hepatic cysts, the possible diagnoses include congenital polycystic disease, congenital hepatic fibrosis or Caroli’s disease. Most hepatic cysts are diagnosed during the third trimester of pregnancy [8]. Liver cysts seem to be more frequent in females and in the right lobe, especially segment IV (which constitutes the bulk of the liver from the fourth to the eighth week of development) [9]. They are not associated with cysts in other organs, and can be completely intrahepatic, partially extra hepatic or pedunculated.

Simple NBICs are rarely symptomatic unless they are large as in our series. In such cases, abdominal distension, feeding difficulties (as in patient 3), respiratory distress can be present and require surgical management within the first weeks. Acute complications have also been described, such as rupture (as in patient 1) or hemorrhage.

Even if large (more than 10 mm in diameter) and/or complicated NBICs are rare, the possibility that these cyst might be symptomatic at birth and require urgent surgical management should be explained during prenatal counseling and guide where the birth will take place (with neonatal intensive care and pediatric surgery).

## Footnotes

**Source of Support:** Nil

**Conflict of Interest:** None declared

